# A face-off between Smaug and Caspar modulates primordial germ cell count and identity in *Drosophila* embryos

**DOI:** 10.1080/19336934.2024.2438473

**Published:** 2024-12-24

**Authors:** Girish Deshpande, Subhradip Das, Adheena Elsa Roy, Girish S Ratnaparkhi

**Affiliations:** a Department of Biology, Indian Institute of Science Education & Research, Pune, India; b Department of Molecular Biology, Princeton University, Princeton, NJ, USA

**Keywords:** FAF1, *Drosophila*, germ plasm, maternal, PGCs, Oskar, Smaug, centrosome

## Abstract

Proper formation and specification of Primordial Germ Cells (PGCs) is of special significance as they gradually transform into Germline Stem Cells (GSCs) that are ultimately responsible for generating the gametes. Intriguingly, not only the PGCs constitute the only immortal cell type but several specific determinants also underlying PGC specification such as Vasa, Nanos and Germ-cell-less are conserved through evolution. In *Drosophila melanogaster*, PGC formation and specification depends on two independent factors, the maternally deposited specialized cytoplasm (or germ plasm) enriched in germline determinants, and the mechanisms that execute the even partitioning of these determinants between the daughter cells. Prior work has shown that Oskar protein is necessary and sufficient to assemble the functional germ plasm, whereas centrosomes associated with the nuclei that invade the germ plasm are responsible for its equitable distribution. Our recent data suggests that Caspar, the *Drosophila* orthologue of human Fas-associated factor-1 (FAF1) is a novel regulator that modulates both mechanisms that underlie the determination of PGC fate. Consistently, early blastoderm embryos derived from females compromised for *caspar* display reduced levels of Oskar and defective centrosomes.

## Introduction

In insect embryos, localized maternal determinants deposited in the egg regulate embryonic development. We recently reported that the *Drosophila* orthologue of human Fas-associated factor-1 (FAF1) encoded by *caspar* (*casp*) is a maternal regulator of PGC specification, and controls total germ cell count [1]. Casp works in conjunction with its interacting partner Transitional endoplasmic reticulum 94 (TER94) [2,3]. Maternal loss of either *casp* or *TER94* results in cytoskeletal abnormalities leading up to defective gastrulation, both phenotypes correlated with aberrant centrosome behaviour. In keeping with the PGC-specific phenotypes, both proteins are enriched in the PGCs. Importantly, a decrease or increase in Casp activity is consistently reflected in Oskar levels, a critical determinant of germ cell identity. Lastly, we identified translational repressor Smaug, an embryonic regulator of *nanos* (*nos*), as a potential critical target of Casp that mediates its PGC-specific function(s). Satisfyingly, our data agree well with the recent observations from the Lipshitz lab, who demonstrate that Smaug functions as a negative regulator of *oskar* translation possibly via its binding to the Smaug Recognition Elements (SREs) within the 3’Untranslated region (3’UTR) of *oskar* RNA [4]. Intriguingly, maternal loss of *casp* or *TER94* also results in defective centrosome separation in dividing pole buds and embryonic PGCs [1]. Here, we discuss these related findings in the context of two determinants of germ cell fate: a) Oskar protein levels and, b) centrosome dynamics.

## PGC formation and specification: a preamble

### Consecutive nuclear division cycles lead to syncytial blastoderm formation prior to cellularization

In *Drosophila melanogaster*, fertilization culminates in the formation of a unicellular embryo that carries a single nucleus resulting from the fusion of the male and female pronuclei, respectively. Unlike other organisms, in the *Drosophila* embryo the nucleus produced post-fertilization undergoes serial nuclear division cycles (NDCs) that consist of G2-M transitions alone (but are devoid of G1 and S phases). The 13 consecutive nuclear divisions occur at a regular time interval (8–10 min at 25°C) in the absence of cytokinesis. These serial mitotic nuclear division cycles give rise to a ‘syncytial blastoderm’ embryo (or a ball of nuclei). Subsequently, it undergoes cellularization during a considerably longer lasting NDC14 [6].

As cellularization is in progress, two landmark events are initiated that together constitute maternal to zygotic transition (MZT) [7,8]. The maternally deposited determinants, including proteins and RNAs undergo selective degradation. As ~65% of the total number of RNAs undergo degradation, genome-wide activation of zygotic transcription (ZGA) is initiated. Consequently, the zygotic determinants control the developmental progression, while the influence of the maternal determinants is gradually diminished. Our recent studies documented a novel involvement of Casp and its protein partner, TER94 [1], in the process. Embryos maternally compromised for either *casp* or *TER94* showed defects in germ band extension which were traced back to the prior stage as these embryos displayed aberrant cellularization [1].

### PGCs are the first embryonic cells to be specified

**Figure 1.**
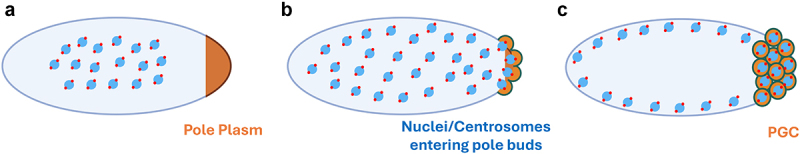
Migration of somatic nuclei into the germplasm and the subsequent generation of PGCs is essential for germline/soma distinction. Around nuclear cycle 8–9 (A), a few somatic nuclei (blue) migrate to the posteriorly localized germ plasm (orange). Individual centrosomes associated with nuclei (red dot) organize microtubule-based spindle assembly (B). Salient observations from [12] followed by [13] together established an essential function of the centrosomes during PGC formation as they demonstrated that centrosomes dissociated from nuclei are sufficient to form germ cells. maternally *deposited oskar*, anchored at the posterior pole of the oocyte (not shown in figure) is essential for germ plasm assembly (orange, panel A) and, subsequently, for PGC formation (orange cells with nuclei, C), as maternal depletion of oskar leads to complete absence of PGCs. In the cellular blastoderm embryos (stage 5) there are 30–32 PGCs at the posterior pole. *figure generated using Biorender.*

The early embryonic development, even prior to cellularization, is marked by three significant developmental events including initiation of (i) early patterning, (ii) somatic sex determination and (iii) germline/soma distinction. Somatic pattern formation involves the subdivision of the embryo controlled by the localized maternal determinants, which regulate their respective zygotic targets, including gap and early pair-rule genes (e.g. *hunchback, even skipped, fushi tarazu, tailless*, among many others) [9]. Somatic sexual identity is regulated by the absolute concentration of zygotically transcribed, X-chromosome linked elements or XCEs (e.g. sisterless-a, scute or sisterless-b, runt) [10,11] that work in conjunction with the uniformly distributed maternal regulators such as Daughterless. While these two events happen around NDC11, germline/soma distinction is established even earlier, when nuclei dividing in the centre of the embryo start their journey towards the nuclear periphery (Figure 1). A few nuclei precociously migrate into the posteriorly localized and anchored specialized cytoplasm or ‘germ plasm’ that contains RNA and protein determinants required for germ cell formation and specification. The recruitment and stepwise assembly of the components of germ plasm depends on Oskar, the critical player shown to be necessary and sufficient for the formation of functional PGCs.

### Oskar is the primary determinant of germline/soma distinction

Oskar is regarded as the ‘master’ determinant of cell fate for PGCs [5] [14,15]. This claim has been substantiated by a host of experiments. In particular, the ectopic presence of *oskar* at the anterior end of the embryo engineered by expressing *oskar-bicoid3’UTR* transgene resulted in the formation of ‘functional’ PGCs at the anterior end of the embryo [14]. This ‘sufficiency’ test underscored the critical status of *oskar* during PGC determination. The Oskar-centric perspective thus proposed that maternally deposited determinants recruited and assembled under the influence of Oskar are sufficient to establish the germline/soma distinction. Surprisingly, however, the crucial contribution of centrosome-dependent transport of germ plasm, and the associated machinery have not received much-deserved attention. In this regard, it is noteworthy that pre-syncytial nuclear division cycles and centrosome function are Oskar independent [16] supporting the idea that two mechanistically separable mechanisms contribute to embryonic PGC formation. In the following, we will revisit some of the early observations and recent data that supports the critical functions centrosomes perform during germ cell formation and specification.

### Centrosomes, and not the nuclei, are also crucial for PGC formation

The pioneering studies carried out by Raff and Glover paved the way to establish crucial function of the centrosomes during PGC formation [12]. Their observations relied upon injection of aphidicolin, a drug that effectively uncouples DNA synthesis and nuclear division from centrosome replication as it selectively inhibits DNA synthesis. Consequently, injection of aphidicolin at nuclear cycle 7–8, resulted in almost complete absence of the normal migration of nuclei to the embryo cortex. In sharp contrast, coordinated cortical migration of the centrosomes occurred as in the case of control embryos injected with buffer. Critically, in such embryos, the centrosomes that ended up at the posterior pole induced PGC formation despite being dissociated from the nuclei. Together, these data argued that centrosomes are capable of initiating the restructuring of the cortical cytoskeleton essential during cell formation. Importantly, PGC formation specifically at the posterior pole strongly suggested that centrosomes ought to play a crucial role in the budding and precocious cellularization of the PGCs. Furthermore, injection of colchicine, which inhibits tubulin polymerization, resulted in nearly complete loss of PGC formation. Taken together, these data suggested that centrosomes and tubulin-based cytoskeleton are essential to induce pole bud formation and PGC cellularization. These findings also raised important issues concerning the nature and transport of critical PGC constituents underlying establishment and maintenance of germline/soma distinction in early embryos. Subsequent live imaging analysis sought to answer some of these questions [13]. As summarized below, their observations confirmed and extended functional significance of centrosomes during germline/soma segregation.

### Centrosomes entering the germ plasm are crucial for PGC formation and specification

Live imaging of germ plasm components (*nanos* mRNA and Vasa protein) was employed to elucidate cell biological and molecular mechanisms underlying segregation and selective transport of germ plasm components [13]. Their data showed that active and regulated transport achieves segregation of germ plasm components in the PGCs. First, centrosomes associated with the posteriorly localized nuclei induce release of germ plasm from the cortex. Subsequently, dynein-dependent transport recruits germ plasm determinants (proteins and RNAs) using centrosome-nucleated microtubular tracks. Importantly, they observed that in the early PGCs undergoing mitotic division, astral microtubules are specifically involved in relatively uniform distribution of germ plasm between daughter cells. Together, these observations strongly suggested that centrosomes are sufficient to release the anchored germ plasm needed for PGC formation and specification. This claim was validated by observing PGC formation in *pangu* (*png*) mutant embryos wherein embryonic nuclei and centrosomes are detached from one another due to severe mitotic defects. And yet an infrequent, accidental entry of a centrosome dissociated from the nucleus, into the germ plasm, is sufficient to induce PGC formation [13].

Together, the microtubule components, dynein, a minus end motor protein, and downstream actomyosin cytoskeletal machinery engineer PGC formation via precocious cellularization [13,17]. Segregation of germ plasm constituents presumably prevents intermixing between the somatic and germline determinants. Furthermore, although germ plasm is anchored to the posterior cortex, it is a highly dynamic entity consisting of complex Ribonucleoprotein particles (RNPs) that are motile which is facilitated by plus- and minus-end motor proteins and microtubules ([18]. Furthermore, proteins that constitute pericentriolar material (PCM), like Pericentrin, also actively participate during PGC formation and subsequent mitotic divisions [19].

Further, emphasizing the importance of the centrosomes during the PGC formation and specification, Lerit et al. subsequently demonstrated that an important constituent of germ plasm ‘Germ cell less’ (or Gcl), modulates centrosome dynamics and thus, contributes to equitable distribution of germ plasm components to the daughter cells [20]. This was particularly relevant as Gcl was initially identified as a maternal component essential for pole bud formation [21]. Consistently, *gcl* mutant embryos show significant loss of pole buds and early PGCs which was attributed to a combined outcome of reduced level of germ plasm and inappropriate activation of mRNA transcription. Surprisingly, broad inhibition of transcription, engineered by alpha-amanitin injections, in young embryos could not rescue pole cell loss in *gcl* mutant embryos [17]. Furthermore, an independent study showed that the Gcl protein contributes to Cullin-mediated protein degradation of the terminal patterning determinant, Torso receptor, in a PGC-specific manner, possibly enabling the pole buds to escape the terminal fate [22]. Of note, PGC specific expression of a degradation-resistant form of Torso indeed mimics the pole bud loss observed in *gcl*^−^ embryos. Based on these data, it was proposed that the role of Gcl protein during the establishment and/or maintenance of transcriptional quiescence in pole buds may not be critical to its phenotype.

While revisiting this issue, [23] observed ectopic activation of the same subset of genes (*sisterless a*, *scute* and *Sxl*) that are activated in the absence of Gcl when a degradation-resistant form of Torso was expressed in the PGCs. Thus, the involvement of Gcl in silencing transcription in the pole buds appeared to be relevant. This was corroborated by the significant rescue of *gcl* phenotype upon simultaneous loss of *Sxl*, one of the important (and likely transcriptional) targets of Gcl. In sum, it remains to be determined if the function of Gcl during pole bud formation is independent of its ability to either directly or indirectly silence transcription in pole buds and PGCs. Nonetheless, together these data argue that both aspects of MZT i.e. protein/RNA degradation and ZGA are regulated in PGCs differentially and likely contribute to PGC determination in a critical manner. Moreover, different germ plasm components perform overlapping functions to establish and maintain germline/soma distinction.

## A versatile new player in town: Casp controls Oskar levels as well as activity of the centrosomes

Taken together, early embryonic development of PGCs goes through multiple steps that are controlled by the assembly, localization, and posterior anchoring of germ plasm, which is regulated by the maternally deposited Oskar. The regulated release of the germ plasm from the posterior cortex is executed by the microtubules emanating from the centrosomes associated with the nuclei that enter the germ plasm at NDC 8/9.

**Figure 2.**
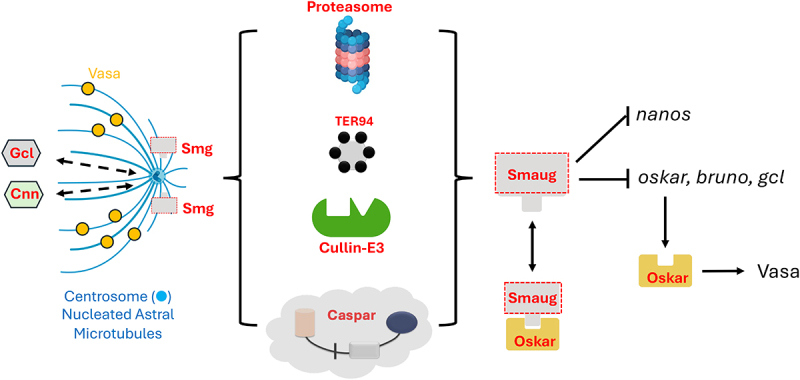
Homeostatic control of Germ-Plasm assembly and PGC count. Both maternal Smaug (smg) and maternal Casp regulate germ cell count post-fertilization [4]; [1]. ‘Loss of Smaug’ or ‘gain of Casp’, leads to excess pole buds and PGC’s. The microtubule network that emanates from the centrosomes that enter the posteriorly localized and anchored germ plasm are critical for the acquisition of germ cell fate [12]; [13]; [20]. Smaug is an Oskar interacting protein [24] and is a negative regulator of the translation of a subset of germ cell components (*oskar*, *bruno* and *gcl* mRnas). Loss of Germ cell less (gcl) also leads to reduced number of PGCs [25]. The Casp/TER94 axis, working with cellular components of protein degradation pathways (shown in brackets, -[]-), likely modulates the degradation of multiple germ cell specific proteins thereby controlling the number of PGCs, during the MZT. The degradation, at least for a significant fraction of targets, may involve the activity of E3 ligases, like the cullin-ring E3 ligase complex that poly-ubiquitinates and marks the proteins for degradation as also terminal degradative machinery, such as the proteasome. Part of the figure was generated using Biorender.

Furthermore, collectively these data suggested that two seemingly independent pathways influence PGC formation and PGC development in early embryos. The first involves precocious migration of the nuclei along with associated centrosomes into the germ plasm, whereas the second depends on the proper assembly and anchoring of the germ plasm to the posterior cortex. Ongoing molecular genetic analysis has uncovered several components that play a critical role in one of the two processes. Our data on Casp are especially novel as it appears to be the only protein component that seems to modulate both the determinants of germ cell formation and/or specification namely Oskar levels and centrosome function/dynamics (Figure 2). Consistent with its influence on Oskar levels, loss of *casp* leads to substantial reduction in total number of PGCs (average numbers from 30 to ~8) formed in the embryo, whereas overexpression of *casp* results in corresponding increase (from 30 to 42) due to extra PGC divisions as reflected in the elevated number of PGCs that are phosphohistone-3 (pH3) positive. As the proper assembly of germ plasm in the oocyte depends on the centrosome function, compromising centrosome activity maternally results in a reduction in the Oskar activity/levels at the posterior of the oocyte. Since Oskar is the critical determinant of germ plasm assembly, it leads to reduction in PGC numbers in the embryo. Thus, a linear pathway triggered by maternal loss of Casp that affects Oskar levels could potentially explain the phenotypic consequences observed in *casp*^*lof*^ embryos. Contradicting this scenario, however, is the observation that loss of *oskar* results in severe reduction in embryonic PGC count, whereas it does not affect nuclear or centrosomal migration adversely in early embryos [16]. *casp*^*lof*^ embryos, on the other hand, display PGC loss as well as nuclear and centrosome migration defects. Together these data argue that Casp may influence two determinants of PGC determination, and we favour the model implying Casp in two independent functions regulating both mechanisms. Additional experiments will be necessary to formally establish that these activities are mechanistically independent, however.

## Duel between Casp and Smaug shapes PGC identity by influencing Oskar levels

Importantly, the reduction in Oskar level corelates with corresponding increase in the Smaug activity in the mutant PGCs. Smaug was initially identified as a negative regulator of *nos* RNA localization and/or translation in the embryos [26,27]. Early reports indicated that activity of Smaug repressed unlocalized *nos* RNA translation to ensure precise posterior patterning, which is primarily mediated by Nos protein gradient. Contemporaneous studies also indicated genetic and biochemical interaction between Smaug and Oskar. The biochemical association between the two proteins is thought to prevent Smaug from binding to *nos* RNA so that it is unable to promote *nos* translation in germ plasm. This is specifically dependent on the presence of the SREs located within the nanos 3’UTR as the binding of Smaug to the SREs via sterile-alpha motif (SAM) domain results in translational repression. Furthermore, Smaug interacts with the Cup protein and eIF4E-binding protein, Cup and blocks the binding of eIF4G to eIF4E [28,29]. Thus, the ability of Smaug to repress translation of *nos* RNA depended on Cup-dependent block in eIF4G recruitment involved in activation of translation.

The ability of Smaug to interfere with the general components of the translational machinery prompted questions regarding a broader function of Smaug during Maternal to Zygotic transition. Subsequent studies established that while Smaug indeed is a specific repressor of *nos* RNA, it is an important participant of a multi-functional RNA Binding Protein assembly critical for maternal mRNA degradation in an embryo-wide manner. A possible underlying mechanism is regulation of miRNAs by Smaug which regulates *miR-3, miR-6, miR-309* and miR-286 during MZT [30]. Supporting the claim, nearly ~85% of mRNAs targeted for degradation remain stable in the absence of Smaug activity. Consistently, Smaug protein accumulates relatively uniformly across the early embryo at the onset of embryogenesis. Presence of a stem-loop structure formed by the SREs allows recruitment of Smaug resulting in either degradation or repression of translation of a variety of maternal transcripts. For instance, Smaug protein forms a complex with translational repressors, including the eIF4E-binding protein, Cup; the DEAD-box helicases ME31B and Belle, and the FDF-domain protein, Trailer hitch (TRAL) [31]. Furthermore, Smaug protein is thought to attenuate mRNA expression via the recruitment of proteins that induce the mRNA decay including the CCR4-NOT deadenylase complex [32,33]. Curiously, at the end of the mid-blastula transition, Smaug protein also undergoes rapid degradation [31]. However, both the functional relevance of Smaug clearance as well as mechanism underlying proteolytic destruction are as yet unclear.

As stated previously, recent data from the Lipshitz lab, however, has shown that Smaug protein also functions as a negative regulator of *oskar* translation likely by directly binding to the SREs located within the 3’UTR of *oskar* RNA [4]. This interesting layer of regulation in fact supports the idea that various homoeostatic mechanisms are likely at play that are essential for establishing the germline soma distinction and interestingly proteins required for functions seemingly dedicated to somatic patterning including ZGA in fact play a critical role in the process. This is reflected in the PGC specific enrichment of Casp and Smaug.

## Possible mechanisms and future directions

Recent data have highlighted reciprocal activities of Casp and Smaug vis a vis Oskar levels and/or stability. In the PGCs Smaug regulates translation of Oskar leading alterations in total PGC count. Using a number of genetic combinations between *smaug* mutants, Lipshitz, Gavis and Smibert labs demonstrated [4] that loss of Smaug in the syncytial embryo leads to excess number of PGCs (increase from 30 to 40) which is reminiscent of Oskar overexpression. Consistently, Siddiqui et al. [4] also showed that absence of Smaug led to increased levels of Oskar (and Vasa). It was thus hypothesized that Smaug’s effect on PGC numbers was a consequence of ‘surplus germ plasm assembly’. These observations underscore the idea that even after germ plasm formation/deposition, in the oocyte, the activity of the germ plasm, in the syncytial embryo can be modulated. Intriguingly, using live imaging [4], demonstrated Smaug co-localizes with Vasa in the PGCs, and a fraction of the protein also appears to be associated with centrosomes [34] had previously showed that translation of *smaug* RNA is regulated by Pan GU kinase). Moreover, while Smaug is actively degraded in the somatic compartment of the embryo, the protein lasts longer in the PGCs.

Casp, a protein initially identified as a negative regulator of IMD/NFkappaB signalling [35], performs an antithetical role as compared to Smaug. Accordingly, embryos derived from *casp*^*lof*^ mothers display fewer pole buds and PGCs, whereas overexpression of Casp leads to excess PGCs (average count 40), comparable to those seen in *smaug*^*lof*^. Since both genes are expressed in the syncytial embryo but not in the oocyte, this suggests that Casp and Smaug have opposing activities, in the syncytial embryo. Furthermore, the disparate functions of the respective proteins likely influence both the pole bud formation and PGC division. Casp associates with its protein interaction partner TER94, a known positive regulator of proteasomal degradation. Casp/TER94 could thus regulate ubiquitin-mediated degradation of Smaug and/or Oskar. [1] showed that *casp*^*lof*^ embryos had excess Smaug, suggesting that Casp’s effect on PGCs was mediated at least in part via Smaug.

Their biochemical data also suggested that in the absence of Casp, Smaug degradation is delayed, perhaps leading to a decrease in the ‘activity’ of germ plasm, which is consistent with Oskar’s reduced translation/activity. By contrast, upon Casp overexpression, Oskar levels rise, and as a consequence, PGC numbers are elevated. As emphasized earlier, in a fly embryo, PGC determination is regulated by the components of maternally deposited germ plasm and centrosome dynamics. What renders Casp activity especially unique and exciting is its functional connection with both the determinants of PGC fate and numbers.

Future experiments will thus focus on elucidating mechanistic underpinnings of how Casp functions to regulate the Oskar protein and activity. Whether this is, in part, controlled by the ability of Casp protein to influence *oskar* RNA localization also remains to be determined. In wild type germ plasm, *oskar* RNA is segregated to the founder granules that are distinct compared to the germ granules consisting of Vasa and Oskar proteins, *nos* RNA, etc. Moreover, forced mis-localization of *oskar* RNA to germ granules results in germ cell loss [36]. Thus, it may be interesting to assess if *oskar* RNA is mis-localized to germ granules upon loss of *casp,* thereby contributing to germ cell loss seen in *casp* mutant embryos.

The disparate functions of Casp and Smaug are of special interest for several reasons, as both proteins are also needed in the somatic compartment for the proper progression of MZT. It remains to be determined whether either protein has any direct influence on the activation of zygotic genome, however. As known determinants of ZGA such as Zelda and CLAMP contribute to the specification of both the soma as well as germline [37], it may be worthwhile to assess potential involvement of Casp and Smaug during ZGA in both these compartments as well.

The recent findings have clearly established that both Smaug and Casp proteins play a crucial role during proper specification of early PGCs, and determination of the final PGC count. What remains to be investigated are the precise mechanisms underlying their functional involvement during localization, translation and degradation of different germ plasm components (RNAs as well as proteins such as Oskar, Nos, Gcl, Vasa, etc.). Understanding the reciprocal regulation of Oskar via Smaug and Casp could serve as an effective handle to elucidate the homoeostasis that establishes and maintains the soma/germline distinction in the early embryos.

Lastly, germ plasm specific homotypic clustering of different PGC-specific RNAs (*nanos* or *pgc* etc.) mediated by the respective 3’ untranslated regions (3’UTRs), is variable between different *Drosophila* species. The differences can be potentially attributed to sequence-specific variation within the 3’UTRs between the species and are correlated with the final ‘healthy’ PGC count in the coalesced gonads [38]. It may be of interest to assess if the Casp/Ter94 axis also contributes to this diversity across the species by influencing the homotypic clustering via its potential ability to modulate the RNA localization, stability and clustering.

## Data Availability

No data was generated or analysed in the article.
